# Reactive oxygen species in leukemias: maintaining cancer cell proliferation via redox signaling and changing metabolic homeostasis

**DOI:** 10.18632/oncotarget.27913

**Published:** 2021-05-11

**Authors:** Andrew J. Robinson, Richard L. Darley, Alex Tonks

**Keywords:** AML, ROS, REDOX, metabolism, glycolysis

Acute myeloid leukemia (AML) is a heterogeneous disease, which typically affects older people and is associated with poor clinical outcomes [1]. Whilst there have been significant improvements in AML therapy in the last few years, survival rates remain low, coupled with high incidence of relapse and therapy resistance [2]. The development of all trans retinoic acid (ATRA) in the treatment of APL (reviewed in [3]) and tyrosine kinase inhibitors (TKI) as targeted therapy in chronic myelocytic leukemia (CML) (reviewed in [4]), provide a paradigm for targeted therapy in AML. Indeed, revelations of novel insights into pathways that lead to the survival and proliferation of AML cells is opening new therapeutic possibilities [5, 6]. One such possible pathway includes reprogramming metabolism. A recent report by our group, Robinson *et al*., 2020, showed that reactive oxygen species (ROS) which are inappropriately produced by NAPDH oxidase (NOX2) on the surface of AML cells, can alter AML cell metabolism to support proliferation [7].

Our previous studies linked increased NOX2 derived ROS production with cellular proliferation in human hematopoietic stem and progenitor cells (HSPC) [8]. Follow up *ex vivo* studies showed similar effects in AML patient blasts and *in vitro* AML cell lines models [9]. Further, other groups have shown that FLT3-ITD AML showed increased levels of ROS which associated with increased DNA double strand breaks [10]. Clinically, a causal link between ROS level and relapse has been established in AML [11], whilst elevated ROS levels have also been observed in ALL and CML patient samples [12]. Building on work by Hole *et al*., 2010 [8], our group found that HSPC expressing constitutively active RAS not only generated increased levels of NOX2 derived ROS, but that this ROS specifically led to changes in mRNA expression levels of glycolytic enzymes [7]. Data presented in this study established for the first time a link between ROS production, increased glycolysis and elevated expression of the key glycolytic regulatory enzyme, 6-Phosphofructo-2-Kinase/Fructose-2,6-Biphosphatase 3 (PFKFB3) in AML patients. Support for these findings was provided using AML cell lines, where ROS levels were depleted through chemical inhibition of NOX or knock-down of NOX2 using shRNA (THP-1) or increased (using lines that generate little NOX2 derived ROS; Mv4;11) through the addition of exogenous ROS [7]. Overexpression of PFKFB3 in Mv4;11 AML cells was sufficient to in increase glucose uptake and cellular proliferation, whilst in cells with activated NOX2, chemical inhibition of PFKFB3 decreased glucose uptake and cellular proliferation in THP-1 cells and HSPC expressing mutant RAS. Together, these data established a causal link between ROS levels, cellular glucose uptake and PFKFB3 activity.

Mass spectrometry was employed to determine the impact of inhibition of ROS production on metabolism in AML cell lines [13]. These data supported the functional changes in glycolysis previously observed; that decreased ROS levels correlated with decreased levels of glycolytic metabolites. Further support for this data was provided by an equivalent experiment using AML patient blast samples, which had been categorised into ROS^High^ and ROS^Low^ producing blasts. Analysis of this data showed ROS^High^ blasts had higher intracellular glucose levels than ROS^Low^ blasts and additionally AML blasts had higher levels of intracellular glucose and lactate than normal hematopoietic (control) cells [7]. Metabolomic data generated as part of our studies [7, 13] also indicated increased levels of ROS correlated with alterations in metabolites linked to FAO and complex lipid homeostasis. NADPH is a crucial electron source for several reductive synthesis reactions, including fatty acids, and nucleotides which may contribute to sustaining tumor cell growth [14]. The generation of NADPH also serves several purposes including managing increased oxidative stress as NADPH is used as a cofactor by glutathione reductase to reduce oxidized glutathione, and by thioredoxin reductase to reduce oxidized thioredoxin. In support of this, upregulation of the transcription factor Nrf2, which regulates gene expression of the peroxiredoxin and glutathione systems, as well as pentose phosphate pathway (PPP) enzymes such as G-6-PD (reviewed in [15]), has been reported in primary cells collected from AML patients [16].

ROS has previously been linked to metabolism, having been shown in endothelial cells to increase HIF-1α expression which in turn upregulates numerous glycolytic enzymes [17]. To try to mechanistically understand how exposure to ROS leads to changes in PFKFB3 expression, our study analysed microarray data for *HIF-1α* expression [7]. The data showed increased expression of *HIF-1α* mRNA correlated with increased ROS levels and given that *PFKFB3* is known to be a target of HIF-1α it was reasoned that changes in expression may be driving PFKFB3 expression. However, HIF-1α is mainly post-translationally regulated and immunoblotting showed that HIF-1α was not expressed at detectable levels and furthermore, knock-down of the mRNA for this protein did not result in any changes in glucose uptake or proliferation. We also investigated the potential role of the stress response kinase p38, which is known to be activated in response to ROS [18]. Further, the promoter region of the *PFKFB3* gene contains a serum response element which has been shown to be activated by the p38^MAPK^ pathway [19]. Whilst activation of p38^MAPK^ by ROS has been shown to occur in our studies (unpublished data), inhibition of p38^MAPK^ did not lead to changes in PFKFB3 expression.

ROS is also known to activate both AKT and AMPK. We showed that inhibition of mitochondrial proteins (UCP2) led to decreased p-AMPK and PFKFB3 levels [7]. AMPK is a master regulator of cellular energy homeostasis, upregulating catabolic metabolic processes including increased glycolytic flux and protects against ROS accumulation by increasing NADPH production. Interestingly, increases in expression of UCP2 has been linked with 2–3 fold elevation of plasma fatty acids reviewed in [20]. Activation of fatty acid metabolism by AMPK (reviewed in [21]) is interesting given that ROS activation of AMPK has also been shown to potentially maintain HSC [22]. Conversely, inhibition of mitochondrial fatty acid oxidation induces loss of HSC maintenance [23].

Whilst our studies clearly established a link between ROS, PFKFB3 expression, cellular glucose uptake and proliferation, several areas warrant further investigation. It is often stated as a key tenet of the Warburg effect, that transformed cells show an increase in aerobic glycolysis, accompanied by increased cellular glucose uptake and lactate secretion. Whilst increases in ROS were shown to increase cellular glucose uptake, corresponding changes in lactate levels were not observed when stratified according to ROS level and the reasons for this have not been delineated. One plausible explanation is that ROS effects changes in monocarboxylate transporter (MCT) or LDH expression, resulting in decreased lactate excretion, or increased lactate uptake and increased conversion of lactate into pyruvate. Given the hydrophilic nature of lactic acid, transport across membranes necessitates transporters that belong to the MCT family encoded by the solute carrier 16 (*SCL16*) family of genes. The main transporter of lactate out of the cell is MCT4 (*SLC16A3*). While MCT4 showed no ROS dependent changes in expression, expression of MCT1 (*SLC16A1*) the main transporter of lactate into the cell was not examined (data not shown). Overexpression of MCT1 has been observed in several cancers and imported lactate can be used for both energy production and biosynthesis [24, 25]. In support of this, a recent study showed that both HeLa cells and the lung cancer cell line H460 use lactate to synthesise lipids during proliferation and as an alternative to pyruvate as a point of entry into the mitochondria and the generation of energy through the citric acid cycle [26]. Furthermore, the process of converting pyruvate into lactate additionally generates NAD^+^, which can then be used to support an increased glycolytic rate.

Lactate may also be retained in the cell to promote the antioxidant response as lactate is known to inhibit PFK activity [27] and thus glycolytic flux. Inhibition of glycolytic flux may result in the diversion of glycolytic metabolites into the PPP, which is a major metabolic pathway in the production of NADPH. Recently it was reported that PPP genes were upregulated in > 60% of AML patients, whilst inhibition of G-6-PD using the inhibitor, 6-aminonictoinamide, inhibited growth and migration of AML *in vitro* [28]. Analysis of metabolomic data presented in the study by Robinson *et al*., is consistent with the hypothesis that elevated ROS results in increased flux through the PPP.

Current treatments in AML appear to have reached their therapeutic limit and the need for either novel stand-alone therapies or adjuncts to traditional chemotherapeutic treatments is clear. There is a growing body of evidence implicating aberrant ROS production and redox signalling in the modification of cellular metabolic pathways, whilst PFKFB3 is known to be dysregulated in numerous cancers. Work by our group has established a causal link between increased ROS production and changes in PFKFB3 expression levels in AML models. However, further work is urgently needed to delineate the full impact of dysregulated ROS production on cellular metabolic function in AML.
